# Down's Syndrome with Alzheimer's Disease-Like Pathology: What Can It Teach Us about the Amyloid Cascade Hypothesis?

**DOI:** 10.4061/2010/175818

**Published:** 2010-06-06

**Authors:** Rania M. Bakkar, Guangju Luo, Thomas A. Webb, Keith A. Crutcher, Gabrielle M. de Courten-Myers

**Affiliations:** ^1^Department of Pathology, MD Anderson Cancer Institute, 1515 Holcombe Boulevard, Houston, TX 77030, USA; ^2^Department of Pathology and Laboratory Medicine, College of Medicine, University of Cincinnati, MSB, 231 Albert Sabin Way, Cincinnati, OH 45221, USA; ^3^Department of Pediatrics and Internal Medicine, College of Medicine, University of Cincinnati, MSB, 231 Albert Sabin Way, Cincinnati, OH 45221, USA; ^4^Department of Neurosurgery, College of Medicine, University of Cincinnati, 231 Albert Sabin Way, Cincinnati, OH 45221, USA

## Abstract

Down's syndrome (DS, trisomy 21) represents a complex genetic abnormality that leads to pathology in later life that is similar to Alzheimer's disease (AD). We compared two cases of DS with APOE *ε*3/3 genotypes, a similar age at death, and comparable amyloid-beta 42 peptide (A*β*42) burdens in the brain but that differed markedly in the severity of AD-like pathology. One exhibited extensive neurofibrillary pathology whereas the other showed minimal features of this type. Comparable loads of A*β*42 could relate to the cases' similar life-time accumulation of A*β* due to trisomy 21-enhanced metabolism of amyloid precursor protein (APP). The cases' significant difference in AD-like pathology, however, suggests that parenchymal deposition of A*β*42, even when extensive, may not inevitably trigger AD-like tau pathology (though it may be necessary). Thus, these observations of a natural experiment may contribute to understanding the nuances of the amyloid cascade hypothesis of AD pathogenesis.

## 1. Introduction

Down's syndrome (DS), caused by an additional chromosome 21, most commonly underlies genetic mental retardation. Alzheimer's disease (AD) is the most common neurodegenerative disease causing dementia. DS is a strong risk factor for dementia that is very similar to AD. DS' prevalence of dementia increases with age: 8% before age of 50, 55% before age of 60, and 75% after age of 60 years. Sporadic AD manifests increasingly after age of 60 years to affect 1 in 4 at age of 85 years [1]. AD-like pathology develops inevitably in DS brains after age of 40 and stages similar to sporadic AD are recapitulated and accelerated in DS brains [2]. The earliest changes in young DS brains (<30 years) consist of intracellular accumulation of amyloid-beta peptide (A*β*) in neurons and astrocytes, followed by deposition of extracellular A*β* with formation of diffuse plaques and, finally, the appearance of neuritic plaques and neurofibrillary tangles [3]. 

The A*β* peptide is a product of two sequential cleavages of the receptor-like amyloid precursor protein (APP). The proteases involved are *β*-secretase (identified as the aspartyl protease BACE-1, the beta-site APP-cleaving enzyme) and *γ*-secretase, a multimeric complex containing presenilin. Alpha-secretase cleaves within the A*β* polypeptide releasing nonamyloidogenic fragments. Gamma-secretase can release either A*β*40 or A*β*42, which has been reported to aggregate more and be cytotoxic. Secreted A*β* can become degraded by metalloproteases or be metabolized through uptake mediated by apolipoprotein E (ApoE) [4–6]. 

The reason why DS patients in later midlife frequently develop dementia and inevitably exhibit AD-like pathology appears to be related to the extra copies of the APP and BACE-2 genes, both located on chromosome 21 in the obligate DS region at 21q22.3, resulting in abnormal processing of APP [7, 8]. BACE-2, an aspartic protease homologous to BACE-1, also cleaves at beta-sites but with a lower affinity compared with BACE-1. While BACE-2 does not play a major role in sporadic AD pathogenesis, its increased expression in DS patients likely plays a more prominent role in causing increased A*β* levels and plaque deposition in this population [7]. BACE-2 is overexpressed in fetal DS brains. It cleaves APP at its beta-site and at a site within A*β* (yielding nonamyloidogenic fragments) [7]. On the other hand, Cheon et al. [8] found overexpression of APP in adult DS brains but described similar BACE-1 and BACE-2 expression to that of controls whereas in early fetal DS brains they found no significant differences in expression. Consequently, life-long deposition of A*β* takes place in DS brains including A*β* *4* *2*, a critical component of amyloid plaque formation that is widely accepted as initiating the amyloid cascade of AD pathology [5, 6]. This view, however, is being challenged [9]. 

According to the amyloid cascade hypothesis of Hardy and Higgins [10], accumulation and aggregation of A*β* is the primary cause of AD, including an inflammatory response followed by neuritic injury, hyperphosphorylation of tau protein, and formation of neurofibrillary tangles (NFTs), leading ultimately to neuronal dysfunction and cell death. This hypothesis of AD pathogenesis may be particularly applicable to DS/AD because of the inevitable development of AD pathology in older DS brains [2] and of DS' abnormalities in APP (and BACE-2) metabolism with life-long overproduction of A*β* [7, 8]. Even so, the present study challenges a central role played by A*β*42 in AD pathogenesis because two similar cases of DS with respect to age at death and the APOE genotype differed markedly in NFTs and senile plaques in spite of having comparable A*β*42 loads.

## 2. Material and Methods

The patients' brain and spinal cord were obtained at restricted autopsy (patient 1) and unrestricted autopsy (patient 2) using standard methods. After a 2-week fixation in 10% buffered formalin, the brain and spinal cord were examined grossly and microscopically. Representative histological sections were obtained from each cerebral lobe, hippocampus, central gray nuclei, brain stem, cerebellar cortex, dentate nucleus, and available spinal cord. Additional sections were obtained from abnormalities including an infarct in case 1. Routine neurohistological sections were prepared at the University of Cincinnati Hospital and additional immunohistochemical studies and determination of the APOE genotypes were carried out at the University of Indiana's Alzheimer's Disease Center. 

Tissue was processed according to standard protocols for embedding the brain in paraffin and each block was stained with hematoxylin and eosin (H&E). Immunohistochemistry was performed with positive and negative controls using antibodies specific for the following:

- *phosphorylated tau* including Tau-5 (Neomarkers, Fremont, CA 94555, pretreated with CC1-mild using Ventana method iVIEW DAB Kit, 1:2000, incubated for 16 minutes at 42°C) and AT8 (Innogenetics, Antwerp, Belgium),
- *a* *m* *y* *l* *o* *i* *d*-*β* (A*β*) (Elan Pharmaceuticals, 1:100) including 10D5 (target antigenic region on the A*β* molecule is peptide 3–6) and 21F12 (target antigenic region on the A*β*-molecule is peptide #42),
- * glial fibrillary acidic protein* (GFAP, DakoCytomation, Carpinteria, 1:100),
- *α* *-synuclein *(*α*-synuclein, Neomarkers, Fremont, CA 94555, prediluted using the Ventana method iVIEW DAB Kit, incubated 32 minutes at 37°C ).

Special stains (with positive controls) included Congo red (Ventana, Tucson, Arizona, using the Ventana method, Congo Red Kit, Nexes Special Stainer) which was viewed under rhodamine fluorescent light to enhance detection of small amounts of fibrillary amyloid, Luxol fast blue/H&E and Woelcke for myelin, Bodian silver impregnation staining axons, and AD pathology and thioflavin S which highlights AD pathology viewed under fluorescent light.

### 2.1. Semiquantitative Assessment of NFT and of A*β*

Loci having either tau or A*β*42 immunoreactivity in either of the two cases were examined. The density of NFTs was assessed semiquantitatively using two IHC stains for tau pathology (AT8 and tau5). Each estimate was carried out within the region of interest showing the apparently highest density of NFT. This region was viewed at a magnification of 400 X and within this field all nerve cells and those with NFT were counted yielding an NFT density expressed as a % of neurons. The results derived with the two IHC methods were then compared by plotting them as a scatter plot with a linear regression line. The 18 data pairs available yielded a significant correlation (*r* ^2^ = 0.6419, *P* <  .001) validating the use of either method's highest NFT density to be entered in Table 1. 

The density of A*β* *4* *2* plaques with 21F12 antibodies and of fibrillar amyloid plaques with the Congo-red method was similarly assessed. Again, within each *β*-amyloid-positive locus the area with the apparently highest plaque density was selected. The number of plaques was estimated at a magnification of 100 X using a modified CERAD scale listed in Table 1. The presence of vascular amyloid was also recorded.

DNA was extracted from cerebellar tissue to determine the APOE genotype using methods described elsewhere [11].

## 3. Results

### 3.1. Clinical History

Case 1 was a 48-year-old Caucasian man with typical features of Down's syndrome, severe mental retardation, superimposed dementia, and spastic gait. For several years prior to his demise, he had developed progressive dementia. He also suffered from generalized tonic-clonic seizures late in the course of his dementia. During the last years of his life, he needed total care. He had swallowing difficulties with a high risk for aspiration, bowel, and bladder incontinence and was wheelchair bound due to bilateral spasticity. On exam, he had no ability to follow commands, but withdrew all extremities to painful stimuli, showed upper gaze palsy, and increased plantar flexion tone. He had full range of movement in both upper and lower limbs and absence of involuntary movements. He had a spastic posture with a high risk for falls. For the last 3 months of life, he received hospice care. An autopsy was restricted to the brain and upper spinal cord. 

Case 2 was a 46-year-old Caucasian man with typical features of Down's syndrome and mild mental retardation. In contrast to case 1, he had not developed dementia as his level of cognitive functioning was stable for at least the last decade and he was able to take care of himself. On exam, he followed commands, he was cooperative, and his neurological examination was within normal limits. During the last year, he had chronic back pain due to intervertebral disc herniation and stenotic fibrosis with compression of the spinal cord for which he underwent a laminectomy and fusion of T7 through L1 vertebrae two weeks prior to death. Following surgery, he recovered without complications, regained his base level of cognitive function, and was transferred to a rehabilitation center. There he developed an acute right common femoral deep venous thrombosis, confirmed by ultrasound exam, for which he was treated. He died two days later. A full autopsy was performed.

### 3.2. Autopsy Findings

Case 1: At autopsy, performed 25 hours postmortem, the body appeared to be one to two decades older than his age of 48 years. He showed typical stigmata of Down's syndrome. The fresh brain weighed 1020 gm (average for gender and age = 1430 gm). The cerebral convexities revealed diffuse cortical atrophy and a cystic infarct, 3 × 3 × 2 cm, affecting the left inferior frontal gyrus anterior to the precentral gyrus. The superior temporal gyri were narrower than normal bilaterally, as is often seen in Down's syndrome. The lateral ventricles were moderately enlarged, with symmetrical dilatation of the frontal, temporal, and occipital horns (hydrocephalus *ex vacuo*). The upper cervical spinal cord revealed no gross pathology. 

Microscopy confirmed a fully organized cortical-subcortical infarct extending into the internal capsule and putamen. The ipsilateral corticospinal tract was mildly atrophied at all levels, including the available upper spinal cord showing myelin pallor of the right (crossed) corticospinal tract. The spinal cord lacked other pathologic changes. Additionally, microscopic intracortical infarcts were found in the frontal lobes. The hippocampus revealed bilateral sclerosis with loss of pyramidal neurons in the CA1/Sommer sector and gliosis. The cerebellar cortex showed foci with Purkinje and granular cell loss and Bergmann gliosis. These ischemic lesions were all located in brain regions with prominent vascular amyloidosis but without other micro- or macrovascular pathology. Cerebral amyloid angiopathy-associated infarcts occur in DS with AD pathology [12]. 

Pathologic changes typical of AD were widespread within the cerebral cortex and also involved subcortical structures. They included granulovacuolar degeneration (hippocampus), neurofibrillary tangles (NFTs), neuropil threads, amyloid and neuritic senile plaques (SPs), and neuronal loss with gliosis. The distribution and semiquantitative density of NFT, A*β*42, and Congo-red-positive amyloid plaques is shown in Table 1. According to CERAD criteria, the high density of senile plaques in widespread neocortical regions indicates a score C and a definite AD diagnosis [13]. NFT in the visual cortex assigns a Braak stage of 6/6 while A*β* plaques in the cerebellar cortex indicate the most advanced stage 5 of A*β* pathology [14, 15]

The APOE genotype in this patient was APOE *ε*3/3. 

Case 2: At autopsy, performed 14 hours postmortem, the body appeared to be the offered age of 46 years. Stigmata of Down's syndrome included, in addition to characteristic facial features, a short neck with webbing, bilateral fusion of the 2nd and 3rd and 4th and 5th toes, and a small brain with a low fresh brain weight of 1080 gm (average for gender and age = 1430 gm) with bilateral smallness of the superior temporal gyri. The brain and spinal cord failed to reveal other gross abnormalities and the ventricular system was normal. Autopsy established as cause of death a saddle pulmonary thromboembolus and bilateral multiple pulmonary emboli with focal hemorrhagic infarctions while the site of a T7-L1 laminectomy was without complications. 

Microscopic CNS examination showed pathologic changes of AD to be limited to the hippocampus, entorhinal, and transentorhinal cortex while deposits of A*β*42 were extensive and widespread. The distribution and semiquantitative density of NFT, A*β*42, and Congo-red-positive amyloid plaques are shown in Table 1 where they can be compared to case 1. Absence of neocortical senile plaques assigns a score of 0 according to CERAD criteria (absence of AD), while the limited distribution of NFT assigns a Braak stage of 2/6 [13, 14].

The APOE genotype in this patient was also APOE *ε*3/3.

## 4. Comparison of the Two DS Cases

Table 1 affords a detailed comparison of the two cases' distribution and extent of NFT, A*β*42 depositions, and Congo-red-positive, fibrillar amyloid plaques. 

A main finding is the two cases' comparable A*β*42-load (as detected by 21F12-immunohistochemistry). The two cases' A*β*42 deposits are extensive and closely similar in the cerebral and cerebellar cortex while subcortical loci are more variable; overall, they are found in 93% of sites in case 1 and in 70% of sites in case 2 and the highest C+ score (defined in Table 1) affects 43% and 39% of sites, respectively, in cases 1 and 2. 

**Figure 1:**
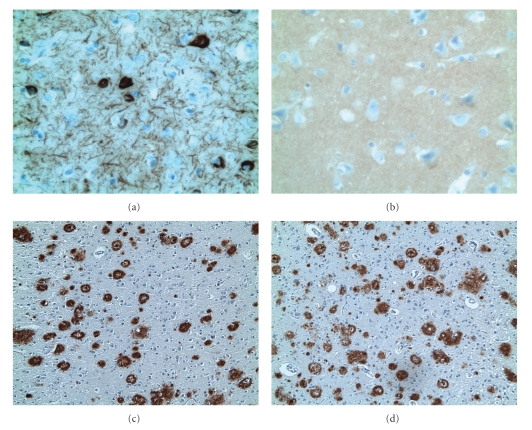
Photomicrographs illustrating the two cases' similarity of amyloid A*β*42 plaques and marked difference in AD-like pathology. Panel (a) shows tau pathology with NFT and neuropil threads of case 1 which are absent in case 2 (panel (b)). Panels (c) and (d) show equally high A*β*42 plaque densities of cases 1 (c) and 2 (d). Location: prefrontal cortex (middle frontal gyrus); immunohistochemistry: tau-5 ((a) and (b)) and 21F12 ((c) and (d)); magnification: 400 X ((a) and (b)) and 100 X ((c) and (d)).

Comparison of A*β*42 in cases 1 and 2 with Wilcoxon's range test for paired observations shows no significant difference between the two cases (*P* >  .10). Yet, in contrast to their similar A*β*42 load, the two cases differ significantly with respect to NFT and fibrillar (Congo-red-positive) amyloid plaques (Wilcoxon's range test for paired observations, *P*  <  .01, resp.). These observations are highlighted in Table 1: yellow shading indicates cases 1/2-data pairs with similar pathology while red shading characterizes those that differ in their extent of pathological changes. The yellow or similar data pairs are prevalent in the A*β*42 column while they are uncommon or absent in the NFT and Congo-red columns. Also, Figure 1 illustrates the two cases' similar A*β*42 plaque density and their marked difference of NFT in the middle prefrontal gyrus.

Analysis of the association of A*β* with NFT shows that all of the 15 Congo-red-positive sites also have NFTs while among the 42 sites with A*β*42 plaques only 28 (2/3) also have NFTs. The number of sites and the density of A*β*42 plaques tend to be higher than those of Congo-red-positive plaques. The locus coeruleus and pontine raphe nucleus stand out for having NFT without A*β* and the cerebellar cortex for diffuse A*β*42 plaques without NFT. 

Alpha-synuclein IHC was negative throughout except for the amygdala where mild focal cytoplasmic positivity was seen on two sections. Of note is that the pigmented nuclei in the brain stem (10th cranial nerve motor nucleus, locus coeruleus, and substantia nigra) are *α*-synuclein negative (nuclei that tend to be affected before or together with the amygdala in Lewy body disease [15]). 

## 5. Discussion

As in the original description of the eponymous disease by Alois Alzheimer, case studies can contribute to mechanistic hypotheses of disease pathology. The present description of two DS cases can be thought of as a natural experiment that could contribute to understanding the complex pathogenesis of AD due to their genetic abnormality with similarly extensive amyloid deposition in the brain but differing in AD-like pathology. The two cases had a similar life span (46 and 48 years), the same APOE genotype (APOE *ε*3/3), and similar distribution and density of A*β*42 plaques but differed markedly in the extent of NFT and Congo-red-positive amyloid plaques: one exhibiting advanced pathology and dementia (case 1) with the other showing only limited, early changes and no apparent cognitive deterioration (case 2). By CERAD criteria, based on Congo-red-positive and tau-positive neuritic (or dense-core) plaques, case 1 received a score C (consistent with definite AD) and case 2 a score 0 (absence of AD) [13]. Using the NFT-based Braak stages, case 1 was at the ultimate stage 6 and case 2 at an early stage 2 [14]. In other words, one case fit the pathological criteria for a diagnosis of AD whereas the other did not.

In DS, AD-like pathology develops in relation to a direct gene dosage effect of trisomy 21 which results in overexpression of APP (and BACE-2) and probably life-long deposition of A*β* [7, 8, 16]. DS is also characterized by premature aging and AD-like pathology at about half the age of sporadic AD. The pattern of NFT and senile plaque formation has been found to be similar in sporadic AD and DS suggesting that they may share the same mechanisms [17]. 

 The similarity in A*β*42 loads in these two cases suggests that the DS-based alteration of APP metabolism with overproduction and deposition of A*β* was similar. This finding argues against the possibility that the two cases differed with respect of full versus mosaic trisomy (without being formerly ruled out). 

Together with the similar A*β*42 loads of the two cases, the marked difference in NFT and Congo-red-positive plaque pathology and difference in dementia may have bearing on the amyloid cascade hypothesis that states that accumulation and aggregation of A*β* is the primary cause of AD pathology [10]. The comparable A*β*42 load found at the same age in our two cases would have been expected to have triggered similar downstream events of NFT, senile plaques, and neuronal loss with gliosis but resulted in extreme differences (at the time of autopsy). Of course, it is possible, even likely, that similar pathology would have developed with additional time in the second case, but the difference in neurofibrillary pathology in the presence of similar amyloid deposition is striking. 

About a decade and a half after the amyloid hypothesis was first proposed, cleavage of amyloid precursor protein to release amyloid-beta peptide has remained a well-accepted hypothesis of AD pathogenesis and has generated multiple therapeutic approaches [18]. However, this hypothesis is not without critique [9, 19] and extensive efforts in targeting the removal of amyloid plaques from the brain of patients with AD have so far been disappointing. Neither plaque-removing vaccines nor the gamma-secretase modulator tarenflurbil have demonstrated clinical benefits [20]. Furthermore, dimebolin (an antihistamine) has been reported to result in clinical improvement in AD while increasing amyloid load [21, 22]. 

Although the present observations question a direct dose-dependent effect of A*β*42 in causing AD pathology, they do not negate the strong evidence from the pathogenic mutations within the genes for APP and presenilins 1 and 2 that are responsible for familial autosomal dominant AD. Similar to DS, these mutations affect APP processing causing overproduction of A*β*42 [23, 24]. However, the possibility that these familial forms of AD cannot be extended to sporadic forms of the disease must be kept in mind.

One of the factors involved in AD susceptibility is apolipoprotein E genotype with *ε*4 (APOE4) being a susceptibility gene and major risk factor for sporadic and familial AD [25, 26]. Also, in DS, the APOE4 allele increases the risk for dementia and, in nondemented adults with DS, those with at least one *ε*4 allele were approximately five times more likely to die within a given followup period than those without [27]. However, since both cases had the same ApoE genotype (*ε*3/3), this cannot account for the marked difference in AD-like pathology. 

 Even if only about half of the sites with NFT also show Congo-red-positive A*β* plaques in the present study, the strong correlation between fibrillar, Congo-red-positive A*β*, and NFT (15/15 sites) could support the view that NFT formation occurs when A*β* is present in fibrillar form [4, 28]. On the other hand, it has been proposed that the A*β* peptides, and especially A*β*42, aggregate in a concentration-dependent manner to form cytotoxic protofibrils that further accumulate to form amyloid plaques [5]. The finding of similar loads of A*β*42 in two DS cases with divergent AD pathology suggests that other factor(s) may play critical roles. 

Ingelsson et al. [29] have shown in sporadic AD that amyloid plaques display a more widespread distribution than NFT and reach a “ceiling” early in the disease process before clinical symptoms become manifest, whereas NFT formation, synaptic and neuronal loss, and gliosis continue throughout the course of AD. Such an early ceiling effect might explain why our DS cases show similar A*β* accumulation in spite of being at early and late stages of AD, respectively. Nonetheless, a trigger mechanism other than A*β*42 accumulation would have to be invoked to have caused progression of AD pathology.

This observation of two comparable DS cases with respect to age at death, APOE genotype, and A*β*42 load does not clarify why the AD-like pathology is so divergent in the two cases, leaving this important question to be resolved by future studies. A possible factor that could underlie the difference in neurofibrillary pathology, in spite of similar overall A*β*42 load, relates to the potential neurotoxicity mediated by prefibrillar or oligomeric forms of A*β*. Recent studies indicate that intraneuronal accumulation of A*β*42 as soluble oligomers may serve as a trigger for progression of AD pathology [30]. Also, only soluble oligomeric but not fibrillar A*β*(1-42) forms induced toxicity in cultured cholinergic cells [31]. To this end, the assessment of intraneuronal A*β* has been rendered easier through development of specific antibodies suitable for immunohistochemistical studies of human postmortem tissue [32]. Such studies may be able to address the still unresolved role of A*β* in Alzheimer's disease.

## 6. Conclusions

This descriptive study of two DS brains with similar age at death and APOE genotype also revealed comparable A*β*42 deposits while one was at an early stage and the other at a late stage of AD-like pathology, suggesting that the mere deposition of widespread A*β*42 is an insufficient explanation for AD progression. Even if A*β*42 deposition may be a necessary link between genetically enhanced APP metabolism and AD development, this limited observation adds to other evidence questioning A*β*42's central role in the amyloid cascade hypothesis of AD pathogenesis. 

## Figures and Tables

**Table 1:** Semiquantitative assessment of AD changes.

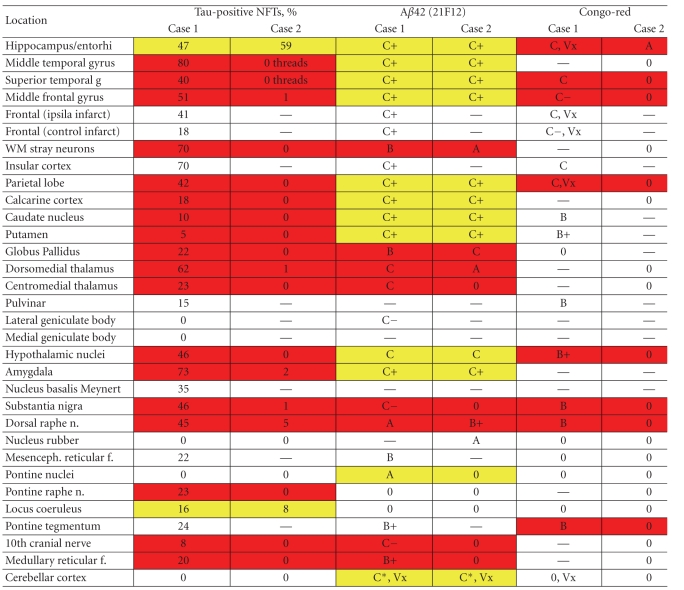

NFTs = Neurofibrillary tangles; expressed as percent of nerve cells affected by NFTs per 400 X field.

Threads = neuropil threads present without NFT.

Amyloid plaque frequency per 100 X field: A = 1–4; B = 5–9; B + = 10–19; C − = 20–29; C = 30–50; C + = >50.

Vx = amyloid-positive vessels.

— = no data; 0 = absent; *large, diffuse plaques in the molecular layer covering ~40% of the area.

Red/yellow shading indicates data pairs with divergent/similar results of cases 1 and 2, respectively.
